# Autoimmune diseases: targets, biology, and drug discovery

**DOI:** 10.1038/s41401-023-01207-2

**Published:** 2023-12-14

**Authors:** Shu-jie Li, Yan-li Wu, Juan-hua Chen, Shi-yi Shen, Jia Duan, H. Eric Xu

**Affiliations:** 1 State Key Laboratory of Drug Research, Shanghai Institute of Materia Medica, Chinese Academy of Sciences, Shanghai, 201203 China; 2 Department of Traditional Chinese Medicine, Fujian Medical University Union Hospital, Fuzhou, 350000 China; 3 University of Chinese Academy of Sciences, Beijing, 100049 China; 4 Zhongshan Institute for Drug Discovery, Shanghai Institute of Materia Medica, Chinese Academy of Sciences, Zhongshan, 528400 China; 5 School of Life Science and Technology, Shanghai Tech University, Shanghai, 201210 China

**Keywords:** autoimmune disease, autoantigen, immunotherapy, type 1 diabetes, insulin, drug discovery

## Abstract

Autoimmune diseases (AIDs) arise from a breakdown in immunological self-tolerance, wherein the adaptive immune system mistakenly attacks healthy cells, tissues and organs. AIDs impose excessive treatment costs and currently rely on non-specific and universal immunosuppression, which only offer symptomatic relief without addressing the underlying causes. AIDs are driven by autoantigens, targeting the autoantigens holds great promise in transforming the treatment of these diseases. To achieve this goal, a comprehensive understanding of the pathogenic mechanisms underlying different AIDs and the identification of specific autoantigens are critical. In this review, we categorize AIDs based on their underlying causes and compile information on autoantigens implicated in each disease, providing a roadmap for the development of novel immunotherapy regimens. We will focus on type 1 diabetes (T1D), which is an autoimmune disease characterized by irreversible destruction of insulin-producing β cells in the Langerhans islets of the pancreas. We will discuss insulin as possible autoantigen of T1D and its role in T1D pathogenesis. Finally, we will review current treatments of TID and propose a potentially effective immunotherapy targeting autoantigens.

## Introduction

AIDs remain a persistent and challenging problem to human health and society, with a prevalence about one in ten individuals [1]. While most AIDs are not monogenic in nature, multiple genetic factors, including specific genetic mutations, HLA diversity, mutations at non-HLA loci, and epigenetic mechanisms, may contribute to their development. In addition, various immune system abnormalities, autoantigenic alterations, microbial infections, environmental factors, gender, and age have also been associated with these disorders.

Unfortunately, diagnosis and treatment of AIDs pose tremendous challenges in clinical practice. The current high treatment burden for AIDs does not adequately address the underlying pathogenic factors driving autoimmunity. Instead, it mainly suppresses symptoms through the use of glucocorticoid hormones and broad-spectrum immunosuppressants, while patients’ immunological function deteriorates as a result of treatment. This highlights the need for more precise targeting and immunomodulation of self-reactive cells, as disease-persistent cells are phenotypically indistinguishable from healthy immune cells, and there are currently no specific immunotherapies for AIDs. In this review, we aim to explain the fundamental mechanistic stages of autoimmune disorders, examine the molecular characteristics and biodistribution of autoantigens, and explore their influence on the immune system. By utilizing these autoantigens as targets, we can gain insights into developing safe and effective autoimmune therapeutics.

## Critical mechanisms underlying AIDs

**Fig. 1:**
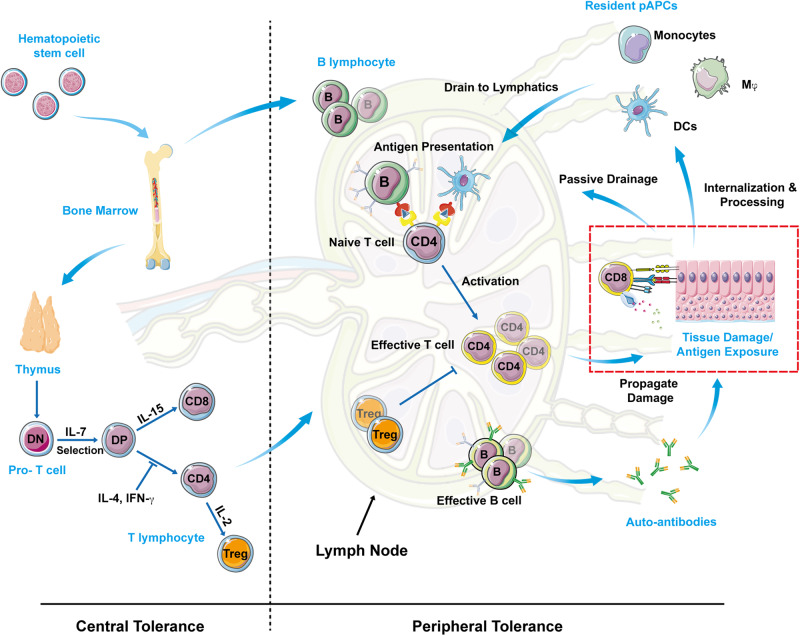
The pathogenesis of autoimmune disease. In normal people, a small number of autoreactive T and B cells “leak out” into the periphery even under the strictest control by central and peripheral tolerance. However, unless there is a genetic predisposition to break tolerance and a trigger or triggers, they will continue to be harmless. Damaged tissue releases autoantigen, which can either go straight to the lymphatics or be taken up by local APCs. Follicular APCs process and deliver antigens to immature T helper cells in the secondary lymphoid organs in the presence of costimulatory signals. The proliferation and differentiation of a cellular or humoral effector response are stimulated in naïve T cells. These activated effectors leave lymphatics and return to antigen-rich tissue compartments, where they might cause more harm and restart the cycle of autoimmunity.

To develop effective antigen-specific approaches for treating aberrant autoimmunity, it is critical to gain a comprehensive understanding of the underlying mechanisms that sustain the disease. Autoimmunity is characterized by a self-perpetuating cycle, wherein a dysregulated immune response is triggered in secondary lymphoid organs and subsequently propagated in tissue compartments that harbor a high density of cognate autoantigens (Fig. 1) [2]. Disruption of tissue integrity, either due to an early insult or persistent damage, can lead to the release of autoantigens, which can then be transported to lymphoid organs via passive or active mechanisms (Fig. 1). This results in the development of an inflammatory microenvironment within the affected tissue, which in turn activates the innate immune system. Sentinel cells, such as dendritic cells (DCs), macrophages (MΦ), and monocytes, are activated and primed to uptake antigens in response to various immune-stimulating signals generated by damaged tissue, such as reactive oxygen species and damage-associated molecular patterns. Although dendritic cells, macrophages and newly arrived monocytes may all uptake autoantigens released from damaged tissues, only dendritic cells can migrate to local lymph nodes, thus being the main carriers of tissue antigens. Active transport of autoantigens suggests their transmission, triggering subsequent migration to nearby lymph nodes. Once autoantigens reach the lymph nodes, they may undergo further processing and presentation to T and B cells in their respective zones [3]. Follicular antigen-presenting cells (APCs) can deliver autoantigens to cognate naïve CD4^+^ T helper cells. It is currently known that naïve T cells are activated in T cell area, but not follicular area (which is B cell area), of the lymph node, by interdigitating dendritic cells that is activated and thus migrating from tissues. If antigen presentation coincides with ligation of surface proteins in the B7 co-stimulatory molecules, then naïve T helper cells become activated. B7 (CD80 and CD86) and CD28 play an important role in the activation of T cells. Proteins CD80 and CD86 on the surface of antigen-presenting B cells and DCs can bind to CD28 on T cells, thereby promoting their activation. These activated cells then multiply and support an effector-driven immune response through receptor and cytokine signaling. The proliferative drive of these activated cells is determined by the T helper subtype as well as cytokines [4]. In nature, antigen-specific effector responses can take various highly-tuned forms, but they can generally be classified into two categories: cellular immunity and humoral immunity. Cellular immunity, also known as cell-mediated immunity, involves the activation of T cells. Naïve T cells are mainly activated by processed cognate antigens presented by major histocompatibility complex (MHC) on the surface of interdigital dendritic cells in lymph nodes. When activated, T cells can recognize antigens presented on target cells, leading to the directed release of cytokines or cytotoxic molecules onto the target cell. Cellular immune responses, such as Th1 and Th17 types, can be distinguished by the inflammatory cytokines they produce. Humoral immunity, involves the production of antibodies by B cells. Antibodies are proteins that recognize and bind to antigens, marking them for destruction by other cells of the immune system. Humoral immunity involves APCs, which are typically activated to engulf target cells or debris in autoimmune disorders. During the effector phase of the immune response, B cells are activated and directed to secrete antibodies by terminally differentiated plasma cells in response to cytokine stimulation. Consequently, the free antigen can be effectively labeled and bound by the development of antibodies. It is now widely accepted that cellular immunity plays a significant role in some autoimmune disorders such as T1D. When effector responses are elicited, these activated cells return to antigen-rich compartments, where they cause autoreactive damage and repeat the production of immunogenic factors, perpetuating the autoimmune cycle (Fig. 1). This pattern of relapsing and remitting is observed in many autoimmune disorders, reflecting the cyclic nature of autoimmune potentiation. Nonetheless, this conceptual framework suggests that antigen-specific immunotherapies can modify the immune system.

## Autoimmune disease classification

Hypersensitivity responses, which are mediated by the immune system, are classified into four distinct types based on the underlying mechanisms of tissue damage. The main autoantigens of various AIDs are outlined in this section, and their potential as therapeutic targets is investigated (Table 1). Type I responses, which are triggered by allergen-specific IgE bound to the high-affinity Fc*ε*R I receptor on mast cells and basophils, can result in the release of various mediators, including histamine, and the development of symptoms such as urticaria, angioedema, and anaphylaxis. Type II responses, on the other hand, are defined by the binding of IgG or IgM to self-antigens on the surface of cells, which can activate phagocytosis, complement-directed cytotoxicity, and/or antibody-dependent cell-mediated cytotoxicity, leading to tissue damage. Type III responses, which are mediated by immune complexes and antigens that lodge themselves in tissues, can directly harm organs. Finally, type IV responses are characterized by delayed reactions mediated by T lymphocytes and can be further subdivided into four subtypes. It is important to note that individual hypersensitivity responses, such as types II, III, and IV, have been linked to autoimmune disorders. However, in clinical practice, hypersensitivity categories may overlap, resulting in patients presenting with a range of symptoms associated with different types of hypersensitivity responses simultaneously.

**Table 1:** Autoantigen targets for common autoimmune diseases.

Disease	Antigen	Interaction domain	Symptoms	Location	Reference
**Autoantibody-mediated AIDs**					
**Type II hypersensitivity reactions**					
Autoimmune hemolytic anemia (AIHA)	Anion exchanger 1(AE1) (band 3)	The C-terminal end of TM 3, the following loop and the loop before TM 8	Anemia	Erythrocyte	[65–67]
Idiopathic thrombocytopenic purpura (ITP)	Glycoproteins (GP)	β-propeller domain	Bleeding	Platelet	[68, 69]
Goodpasture syndrome (GPS)	Type IV collagen (COL4)	α3NC1 and α5NC1 monomers	Glomerulonephritis, pneumorrhagia	Glomerular basement membrane (GBM)	[70]
Grave’s disease (GD)	Thyrotropin receptor (TSHR)	Extracellular domain (amino acids 1–260; TSHR260)	Hyperthyroidism	Thyroid cells	[71]
Myasthenia gravis (MG)	Nicotinic acetylcholine receptors (nAChRs)	MIR, loop alpha1 67–76 in combination with the N-terminal alpha helix alpha1 1–14	Progressive myasthenia	Postsynaptic membrane at the neuromuscular junction	[72, 73]
Pemphigus diseases (PD)	Desmoulins (Dsg3 and Dsg1)	Amino terminal ectodomains of Dsg1/3	Cutaneous herpes	Keratinocyte	[74, 75]
Neuromyelitis Optica spectrum disorders (NMOSD)	Aquaporin 4 (AQP4)	Orthogonal arrays of particles (OAP)	Myelitis/Optic neuritis	Astrocytes	[76, 77]
Thrombotic thrombocytopenic purpura (TTP)	ADAMTS13	Cysteine-spacer domain	Blood clots	Small blood vessels	[78, 79]
Anti-NMDA encephalitis	Heterotetrametric ionotropic glutamate receptor (NMDAR)	Amino terminal-domain of the GluN1 subunit	Psychiatric symptoms	Neuron	[80–82]
Systemic sclerosis	Several, centromere (ACA), angiotensin II type 1 receptor (AT1R), endothelin-1 type A receptor (ETAR)	Several	Fibrosis	Small arteries	[83]
Sjögren’s syndrome	Several, Ro52/TRIM21, Ro60/TROVE2, La/SSB	Several, RING domain, central cavity of the ring	Dryness	Salivary gland epithelial cells (SGEC)	[84–86]
Primary biliary cirrhosis (PBC)	Mitochondria (AMA)	E2 subunits of the 2-oxo acid dehydrogenase complexes (PDC-E2)	Destruction of intrahepatic bile ducts	Cholangiocytes	[87, 88]
Inflammatory bowel diseases (IBD)	Abnormal absorption of bacterial products and/or food antigens	Several	Inflammatory conditions of the colon and small intestine	Intestinal mucosa	[89]
Stiff person syndrome (SPS)	Glutamic Acid Decarboxylase 65 (GAD65)	C-terminal, N-terminal, aa 4–22	Progressive spasmodic muscular	Neurons	[90]
**Type III hypersensitivity reactions**					
Systemic lupus erythematosus	Several, dsDNA, Nucleosomes, Ro, NMDA receptor, C1q, Phospholipids	Several	Symptoms vary among people	Multisystem damage	[91]
Ankylosing spondylitis (AS)	HLA-B*27	Three Ig domains and long cytoplasmic tail 2 (KIR3DL2) receptor	Long-term inflammation of the joints of the spine	Entheses	[92–94]
ANCA-associated vasculitis	Myeloperoxidase (MPO), proteins leukocyte proteinase 3 (PR3)	Active site, A1AT,	Small-vessel vasculitis	Neutrophils	[95, 96]
Antiphospholipid syndrome	β2-glycoprotein-I (β2GPI)	Domain I	Thrombosis	Blood circulation system	[97, 98]
IgA vasculitis (Henoch-Schonlein purpura)	N-acetyl galactosamine (GalNAc)	Gd-IgA1	Vasculitis	Endothelial cells	[99, 100]
Primary membranous nephropathy	M-type phospholipase A2 receptor (PLA2R), THSD7A	CysR, CTLD1, CTLD7, and CTLD8 domain/ sTHSD7A, IDS2, IDS9 and CTE site	Nephrotic syndrome	Podocyte	[101, 102]
**Autoreactive T cell-mediated AIDs**					
**Type IV hypersensitivity reactions**					
T1D	Glutamic Acid Decarboxylase 65 (GAD65), IA-2, insulin, ZnT8, tetraspanin-7	C-terminal (rare), aa 161–243, 473–555/ Core Cysteines, (C909) of Islet Antigen-2 / Insulin B:10–23/ B:12–20/ ZnT8: COOH-terminal(aa268-369)	Hyperglycemia	β-cell	[103–107]
Multiple sclerosis (MS)/Experimental autoimmune encephalomyelitis (EAE)	Myelin basic protein (MBP), myelin oligodendrocyte glycoprotein (MOG)	The double phenylalanine motifs (F4,5 in the amphipathic helix amino acids 85–92)/myelin sheaths	Neurological symptom	Oligodendrocyte	[108–110]
Rheumatoid arthritis	Fc-tail of immunoglobulin (Ig)-Gs/ carbamylated protein (CarP)/ citrullinated protein	IgG-fc domain/ citrulline/ Homo citrulline	Synovial inflammation	Synoviocytes	[111]
Bechet’s disease (BD)	HLA-B*51, CTDP1	MICA transmembrane region (MICA-TM)	Vasculitis	Vascular endothelium	[112–114]
Autoimmune Addison’s disease (AAD)	HLA-DQB*1, HLA-DQA*1, 21-OH, 17-OH and SCC	A tyrosine at the 30th position and an alanine at the 57th position of HLA-DQB1/ an arginine at the 52nd position of HLA-DQA1 / reductase binding site	Adrenalitis, hypoadrenalism	Adrenocortical cells	[115–117]
Autoimmune pancreatitis (AIP)	Several, lactoferrin (LF), CA-IIAb, CA-IVAb, PSTI, trypsinogens, amylase alpha, HSP10, and PBP	Several	Pancreatitis	Pancreatic acinar cells	[118, 119]

In conjunction with the aforementioned pathological mechanisms, we have developed a conceptual framework for classifying AIDs based on whether the autoantibodies in question target extracellular or intracellular proteins, or both. While intracellular autoantigens are typically inaccessible to autoantibodies, they serve as indicators of abnormal immune cell activity and thus, autoimmune disease. Conversely, autoantibodies directed against secreted proteins, cell surface channels, and receptors have easy access to extracellular antigens. Autoantibodies targeting extracellular proteins have the potential to cause disease directly by modifying protein function or abundance, as well as by recruiting complement-mediated cell death. Therefore, we classify autoimmune disorders based on the presence of intracellular or extracellular autoantibodies, which are expressed in distinct tissue regions and serve as disease biomarkers, providing critical guidance for drug development. Many rheumatological disorders, such as Sjögren’s syndrome, systemic lupus erythematosus, systemic sclerosis, and myositis, are characterized by intracellular autoantibodies. Although these autoantibodies do not directly cause illness, they contribute to disease pathogenesis by participating in immune complex formation, complement activation, and immunological activation. We propose that the emergence of intracellular antigens is a consequence of exposure to damaged cells, but their initial source remains outside the cell. Conversely, autoantibodies targeting extracellular proteins may directly induce autoimmune disorders and loss of function. Pathogenicity is determined by the presence of autoantibodies in patients with the disease, the presence of autoantigens in diseased tissue, and the correlation between autoantibody levels and disease activity [5]. Autoantibodies against circulating hormones, growth factors, and cytokines can lead to various autoimmune-mediated disorders. Cytokines play a critical role in regulating immune responses and directing the development of immune cells. Autoimmune conditions with extracellular antigen predominance include myasthenia gravis, membranous nephropathy, and graves’ thyroiditis. It is essential to note that autoantibodies targeting disease-specific target proteins are the primary drivers of clinical manifestations. Monitoring serum levels of pathogenic autoantibodies can provide a more direct way to track treatment responses, as the disappearance or decrease of circulating autoantibodies often correlates with a cure or remission.

## Autoantigen as promising targets for drug developing

**Fig. 2:**
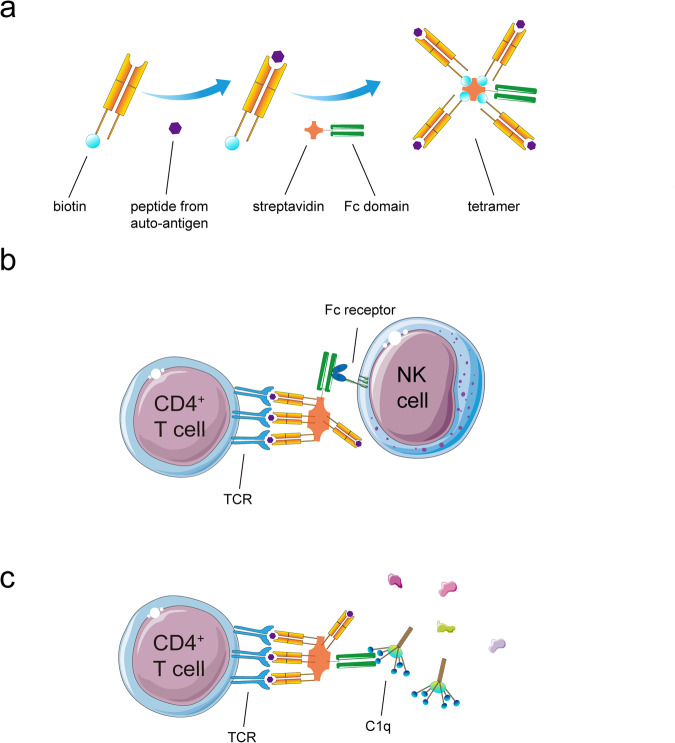
Hypothesis of autoantigen-based therapy for autoimmune diseases. **a** Construction of tetramers about peptide-MHC binding Fc domain complexes. **b** NK cells recognize the antigen-specific T and B cells by pMHC-Fc complexes. **c** pMHC-Fc complexes activate the complement system,which causes the antigen-specific T and B cells to lyse and die by the membrane attack complex.

Autoimmune diseases are pathologically characterized by aberrant immune responses mounted against self-antigens, which leads to chronic inflammation and gradual damage of tissues. A critical step in the pathogenesis of autoimmunity is the activation and proliferation of antigen-specific T and B lymphocytes that recognize self-antigens as foreign. To specifically target these pathogenic antigen-specific T and B cells, innovative therapeutic approaches utilizing complement-dependent cytotoxicity (CDC) and antibody-dependent cellular cytotoxicity (ADCC) have been developed. CDC involves the use of monoclonal antibodies engineered to bind specific antigens present on the surface of target T and B cells. Upon binding its target antigen, the monoclonal antibody activates the complement cascade, ultimately resulting in the formation of the membrane attack complex that causes lysis and death of the bound cell. ADCC utilizes monoclonal antibodies to engage natural killer cells, a type of cytotoxic lymphocyte, to recognize and kill target T and B cells in an antigen-specific manner. Upon recognizing the monoclonal antibody bound to the target cell surface, natural killer cells release perforins, granzymes and other cytotoxins that induce apoptosis of the target cell. Chimeric antigen receptor (CAR) T cell therapy is another promising and innovative strategy to eliminate antigen-specific autoreactive lymphocytes, wherein patient T cells are genetically modified to express synthetic CARs that can recognize specific target antigens on pathogenic B and T cells, thereby inducing cytotoxicity upon binding. Our research team has also proposed a novel immunotherapy using aspects of both CDC and ADCC to specifically deplete antigen-specific T and B cells involved in autoimmunity. We have engineered tetrameric complexes comprising an antigenic peptide-MHC component that confers antigen specificity by binding T and B cells, along with an antibody Fc domain that recruits immune cells to mediate cytotoxicity. The Fc domain can activate natural killer cells through Fc receptors to release cytotoxins, as in ADCC, and can also activate the complement cascade as in CDC, leading to the formation of membrane attack complexes. This dual strategy results in efficient antigen-specific cytotoxicity against autoreactive T and B cells (Fig. 2). In summary, these innovative treatment approaches represent promising strategies to eliminate disease-causing autoreactive lymphocytes in autoimmune diseases, warranting further in-depth investigation to evaluate their safety, efficacy and therapeutic potential.

## T1D pathogenesis: dysregulated T cell activation and autoantibody production

**Fig. 3:**
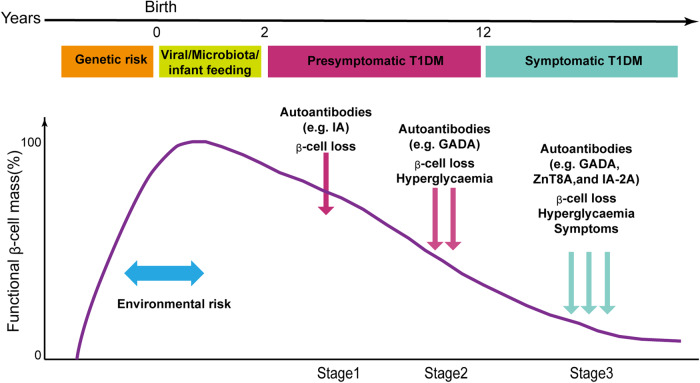
Risk factors and disease progression stage of T1D. Autoimmunity is the outcome of a complex interplay between genetic predisposition and environmental risk factors. Once autoimmunity has been established [stage 1, autoantibodies (particularly those targeting insulin or GAD65) detected with euglycemia], the illness has begun and will inevitably lead to β cell loss, which will in turn damage the capacity to manage glucose (stage 2 dysglycemia), and eventually levels of glycemia diagnostic of T1D and the requirement for insulin (stage 3). (Modified from the Ref. Dayan CM et al., *Science*, 2021).

T1D is an autoimmune disease that results in the destruction of pancreatic β-cells within the islets of Langerhans by the immune system. It was estimated that there were 8.4 million individuals worldwide with T1D in 2021, and this number is expected to rise to 17.4 million by 2040 [6]. Unfortunately, inappropriate treatment and misdiagnosis have resulted in 3.1 million individuals dying prematurely from T1D in 2021 [7]. The incidence of T1D is highest between ages 12–14, likely due to early diagnosis, especially in high-incidence countries [8]. It is concerning that the incidence of T1D is increasing, and there is a high rate of misdiagnosis and inappropriate treatment methods. T1D manifests in three stages (Fig. 3), with clinical signs and hyperglycemia appearing in the later stages [9]. Symptoms of T1D often increase rapidly, with adults experiencing symptoms for an average of 7-8 weeks and children experiencing symptoms for half that time compared to Type 2 diabetes [10].

### T1D pathogenesis

Standardized registry data have shown a 3.4% increase in the incidence of T1D over the past three decades, highlighting the significance of environmental factors in the development of the disease [11]. While various factors have been linked to T1D, none of them alone can account for the rapid rise in incidence. T1D is primarily determined by genetic predisposition, with HLA class II genes, particularly HLA-DRB*103-DQA*105-DQB*102 (DR*3-DQ*2) and HLA-DRB*104-DQA*103-DQB*103:02 (DR*4-DQ*8), as well as HLA class I genes (HLA- A*24, HLA- B*18, and HLA- B*39 alleles), playing a key role. Other genes, such as INS, PTPN22, IFIH1, and CTLA4, located outside the HLA region, also contribute to genetic susceptibility [12, 13]. However, around 90% of individuals diagnosed with T1D do not have a positive family history [14, 15]. Therefore, a new phase of T1D screening aims to delay or prevent clinical disease. Genetic screening can be combined with antibody screening, beginning with GADA and IAA, followed by ZnT8A and IA2A testing for those who test positive, resulting in a cost-efficient approach to screening [16, 17].

Epidemiological and experimental data suggest that enteroviruses, particularly coxsackievirus B (CVB), may contribute to the development of T1D. The presence of CVB in the pancreas can lead to chronic inflammation, which activates the innate immune system, resulting in insulitis and gradual autoimmune destruction of β-cells by pre-existing autoreactive cytotoxic T lymphocytes in genetically susceptible individuals. Additionally, the persistence of CVB in other locations, such as the gut, blood cells, and thymus, may act as a reservoir for pancreatic infection or reinfection, or disrupt central tolerance, leading to islet autoimmunity and T1D [18]. Recent research has shown that destruction of pancreatic β-cells may accelerate the development of diabetes in individuals with COVID-19 prior to the onset of T1D [19]. Epidemiological studies have also indicated an increased risk of developing diabetes among COVID-19 carriers [19]. Dysbiosis of the gastrointestinal tract, caused by alterations in intestinal microbiota and an increased ratio of Bacteroidetes to Firmicutes, has also been associated with the onset of T1D [20]. Although modest associations of unclear significance have been found in some studies, environmental factors such as changes in microbiome, diet, probiotics, household exposures, infections, and geographical location may provide clues to explain the variable kinetics of T1D progression and the ways in which lifestyle interventions can reduce overall risk [12, 21–23].

**Fig. 4:**
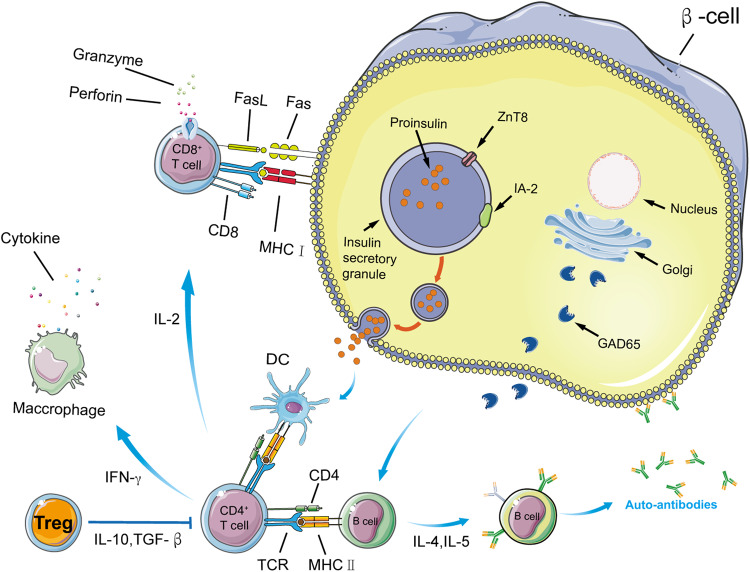
Mechanism of β cell death mediated by T cells in T1D. Proinsulin (PI) has been identified as the β-cell Ag initiator. Insulin granule release may enhance insulin autoantigen exposure, leading to epitope-specific T cell-mediated death of beta cells and T1D. Beta cell death leads to increased release of autoantigens, which in turn stimulates a stronger T cell-mediated immunological response. Pathogenic T cells are activated when APCs migrate from the pancreas, pick up and present diabetogenic auto-antigens, and then travel to the pancreatic islets, where they begin the process of β-cell death and, ultimately, T1D. T_regs_ release cytokines (IL-10 and TGF-β) to protect β-cells from harm. Beta cell destruction also involves autoreactive B cells. To make matters worse, autoreactive T cells then send assistance signals to autoreactive B cells (IL-4 and IL-5), which in turn promote autoreactive B cell development into plasma cells and the generation of islet-specific autoantibodies, thereby perpetuating the vicious cycle that characterizes T1D.

The immunopathogenesis of T1D is characterized by the breakdown of self-tolerance, leading to T cell-mediated destruction of β-cells and the production of autoantibodies (Fig. 4). The pancreas in T1D is characterized by the loss of cells and islet inflammation [24]. Autoimmunity can be activated at any time during development, as evidenced by the identification of autoantibodies, which can occur months to years before the onset of clinical symptoms. Early investigations suggest that younger children experience more severe β-cell loss. Beta-cell loss occurs gradually over time, often before the onset of clinical symptoms, with approximately 90% of cells destroyed by the time symptoms appear [25]. The loss of β-cells in T1D is widely believed to be mediated by autoreactive T lymphocytes [26]. The T1D pancreas is characterized by enhanced expression of HLA class I molecules in the cytoplasm and on the surface of islet cells, which was first reported by Bottazzo and Foulis in the mid-1980s [27]. Both T cells and B lymphocytes play a role in the pathogenesis of T1D, with cytotoxic CD8^+^ T cells being the predominant lymphocyte population. Studies by nPOD have shown that at least some CD8^+^ T cells are autoreactive and target beta-cell autoantigens [28]. B lymphocytes, MΦ, and CD4^+^ T cells are frequently found in insulitis lesions in the pancreas, indicating the involvement of the immune system in T1D [29]. Recent research has shown additional pathological abnormalities in the pancreas, including changes in the extracellular matrix. Hyaluronan (HA), a component of the extracellular matrix, and HA-binding proteins accumulate in the peri-islet area of inflamed islets [30]. HA deposits are observed around capillaries at the edge of diabetic islets, where leukocyte infiltrates occur, as well as within the microvessels of the islets. These pathological changes are chronic and persist for several years after diagnosis and, to some extent, even before diagnosis. Notably, insulitis, β-cell loss, and hyperexpression of HLA class I molecules do not affect all islets simultaneously.

### Autoantibody production as a biomarker of T1D and implicated antigens

The detection of circulating autoantibodies to islet autoantigens has been found to be the most accurate predictor of future T1D, with almost 95% of individuals who exhibit clinical signs of the disease also testing positive for autoantibodies [31]. In longitudinal studies of large birth cohorts with a heightened genetic risk for T1D, there has been a noted peak in islet autoimmunity among children aged 2 to 5 years old, with a more rapid progression to clinical illness observed in these young individuals compared to those who develop the disease at a later age [32–34]. Individuals with autoantibodies against multiple islet autoantigens have been observed to have the highest risk of developing T1D, with approximately 40% of individuals developing T1D within 5 years, 70% within 10 years, and 85% within 15 years [33]. On the other hand, those with only a single autoantibody exhibit a much lower risk, approximately 5%–10%, even with long-term follow-up. Despite the fact that T cells are responsible for the destruction of β-cells, the development of autoantibodies is a good indicator of the disease and typically precedes the clinical onset of T1D. The development of targeted immunotherapies is crucial for the treatment of T1D. To achieve this, it is essential to understand which β-cell derived antigens are responsible for causing T1D and how cognate T and B cells bypass tolerance checkpoints.

Recent research has identified insulin (IN), glutamic acid decarboxylase (GAD), tyrosine phosphatase-like islet cell Ag2 (IA-2), and zinc transporter 8 (ZnT8) as molecular targets of human autoreactive T cells and/or antibodies [17, 35, 36]. This knowledge is critical for the development of targeted immunotherapies for the treatment of T1D. In autoimmunity, the epitope spreading theory suggests that a primary self-antigen (Ag) triggers disease, and the targeting of this Ag leads to tissue damage and the release of additional self-Ags, which perpetuates the autoimmune process. In the NOD mouse, proinsulin (PI) has been identified as the initial β-cell Ag that initiates the autoimmune response [37]. While there is some overlap between the B and T cell antigen targets of T1D in both mice and humans, these targets do not correspond exactly [38]. CD4^+^ T cell epitopes originating from the insulin B chain [39], chromogranin A [40], and islet amyloid polypeptide have been identified [41]. Examples of CD8^+^ T cell epitopes include peptides generated from preproinsulin [42, 43], IGRP [44], zinc transporter 8 [45], and glutamic acid decarboxylase 65 [46]. This understanding of the specific targets of the autoimmune response is crucial for the development of targeted immunotherapies for T1D. A specific peptide, derived from amino acids 10–23 of the insulin B chain, holds significant importance in both the NOD mouse model and in the context of human disease (InsB10:23). This peptide is required for the development of autoimmune diabetes in the NOD mouse model [39], and pancreatic islet autoantibodies (AAbs) against insulin (IAA), glutamic acid decarboxylase (GADA), islet antigen 2 (IA-2A), and zinc transporter 8 (ZnT8A) are produced by B cells and represent the most reliable biomarker currently available for predicting T1D [12]. While some of these targets, such as insulin, IGRP, and ZNT8, are restricted to specific cell types, others, including GAD65 and IA-2, are expressed in a wide range of cell types and organs, including neuroendocrine cells [47]. Defective ribosomal products (DRiPs) and hybrid insulin peptides (HIPs) are emerging as potential initial autoantigens in T1D. DRiPs arise from errors or defects in pre-proinsulin translation [48], while HIPs result from post-translational fusion of proinsulin peptides with other proteins within the secretory granule of β-cells [49]. These epitopes are gaining attention as potential early targets for the immune system in T1D.

### Strategies for preventing and treating T1D

Due to the progressive loss of insulin secretion in T1D, patients often require lifelong treatment with exogenous insulin to maintain glucose homeostasis [50]. However, insulin therapy has limitations in achieving optimal glycemic control, and the risk of hypoglycemia remains a significant barrier to achieving tight glycemic control. Furthermore, studies suggest that peripheral insulin infusion leads to higher circulating insulin levels than portal delivery, which may have long-term implications [51]. Therefore, continued research into β-cell replacement therapies is warranted to address the limitations of insulin therapy and improve glycemic control in T1D patients. In addition to exogenous insulin therapy, islet transplantation has also been attempted as a β-cell replacement therapy for T1D patients. However, the success rate has been limited, with only 12% of patients achieving prolonged graft survival after a single transplant and requiring additional transplants to maintain adequate insulin levels [52]. Along with β-cell replacement therapies, recent advancements in our understanding of β-cell development, glucose metabolism, and the immune pathogenesis of T1D have led to the development of immunotherapies targeting the underlying immune system dysregulation leading to β-cell destruction.

**Fig. 5:**
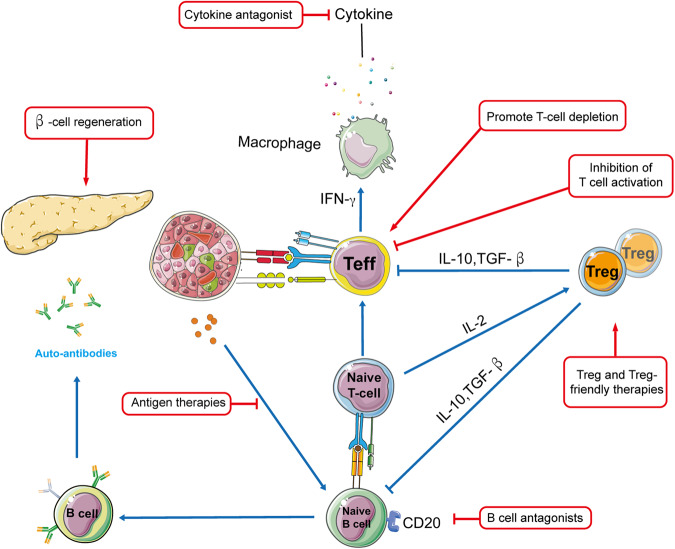
Treatment strategies for type T1D. Multiple immune cell types, routes, and the islets themselves all play a role in the pathophysiology of T1D. T cell (both CD4^+^ and CD8^+^) activation and functioning are shown as the central hubs of the process, since they are responsible for the first stages of β cell elimination in the diagram (solid lines and arrows). Different therapy methods (discussed in detail in Table 2) may be directed at various immune system nodes (shown by boxes) to prevent and cure autoimmune symptoms and β cell destruction. Treatments that suppress pathogenic responses (in red) or boost immune regulation (in blue) have the outcomes shown by the arrows and boxes in this diagram. (Modified from the Ref. Bluestone JA et al., *Science*, 2021).

Given the complex and variable nature of the autoimmune response in T1D, personalized treatment based on individual disease trajectories is crucial (Fig. 5). In this review, we assess the current state of next-generation immune therapies and treatments that have shown promise in type 1 diabetic patients (Table 2). The most advanced immunotherapeutic for T1D currently is teplizumab, an anti-CD3 monoclonal antibody that has been humanized and modified to be nonbinding to Fc receptors. It is currently being evaluated in stages 2 and 3 of T1D clinical trials [53]. Results from clinical trials suggest that teplizumab has shown promise in treating T1D. In patients with T1D at stage 2, teplizumab has been demonstrated to prevent or delay the onset of clinical diabetes by at least 2 years compared to placebo [54]. Additionally, selective T-cell immunotherapies have been successful in patients with T1D at early stage 3. For instance, Abatacept, a selective T-cell co-stimulation modulator, has been shown to slow down β-cell functional decline and reduce HbA1c levels in newly diagnosed individuals who received continuous treatment. Moreover, these benefits were maintained for an additional year after stopping the medication [55]. These results suggest that personalized treatment based on a patient’s specific disease trajectory may be necessary for treating T1D, and next-generation immune treatments and therapies may play an important role in achieving successful outcomes. Abatacept has been found to preferentially affect newly activated autoreactive T effector memory (TEM) cells that are not resistant to costimulation. It achieves this effect through its impact on follicular helper T (Tfh) cells, as shown by mechanistic investigations [56]. This treatment has been shown to lead to improved β-cell function and a decrease in hypoglycemia, which is a common side effect of insulin administration in stage 3 of T1D [57]. Further analysis revealed that Abatacept treatment resulted in a depletion of TEM cells and a selective maintenance of naïve T cells and regulatory T cells (T_regs_).

**Table 2:** T1D drug development at present.

Target pathways	Target	Limitations	Clinical status	References
Inhibition of T cell activation	Anti-CD3, anti-CD2, etc.	Broad suppression induces adverse events	Approved by FDA	[53, 54, 57]
B cell antagonists	Anti-CD20	Not universal or persistent	Approved in multiple AIDs	[58]
Cytokine antagonists	IL-1, TNF-α, etc.	Focus on the last, more advanced stages of illness pathogenesis rather than their earliest stages; recurrent treatment; off-target effects	Several being tested in phase 1,2 studies of stage 3 T1D patients	[59, 60]
Cytokine agonists	IL-2, etc.	Off-target effects; recurrent treatment	Being tested in phase 1, 2 studies of stage 3 T1D patients	[61]
Autoantigen therapies	Peptides, nanoparticles, etc.	Mostly disappointing results	Early clinical trials	[62]
Adoptive cell therapy	CAR/TCR-Treg, etc.	Challenging drug development	Preclinical and early phase 1 trials underway	[63, 64]
β cell regeneration	Islet β cells, pancreatic α cells, etc.	Current therapeutic medicines have limited β cell recovery efficiency	Being tested	[120]

B cell-targeted treatments have shown potential in the treatment of autoimmunity, as they can eliminate both memory B cells and temporary plasmablasts. In NOD mice, reducing pro-inflammatory cytokine production and antigen presentation through B cell reduction with anti-CD20 therapy or genetic disruption of B cell formation prevented the development of diabetes. In T1D stage 3 patients, rituximab, an anti-CD20 antibody, was evaluated and found to preserve C-peptide levels in the short term. However, like T cell-directed treatments, its benefits were neither universal nor long-lasting [58].

Despite efforts to target inflammatory mediators, the effectiveness of such therapies in treating T1D has varied. Clinical studies have shown that canakinumab (a human anti-IL-1β monoclonal antibody) was ineffective in blocking IL-1Ra [59]. In contrast, blocking TNF-α (using etanercept or golimumab) in newly diagnosed T1D patients was associated with a slowed loss of C-peptide, suggesting a potential role for this pathway in T1D [60]. However, in one phase 1/2 clinical study of early stage 3 patients, IL-2 therapy temporarily decreased C-peptide responses, which was linked to an increase in activated NK and CD8^+^ T cells [61].

Immunotherapies aimed at curing T1D have reached a critical juncture. While immunologic tolerance has not proven to be long-lasting, researchers are still striving to identify novel approaches to antigen delivery that avoid triggering an immune response. Traditional methods such as oral insulin delivery and immunization with GAD65 have yielded limited success. In pursuit of more effective strategies, researchers are turning to biosynthetic approaches using nanoparticles, coupled red blood cells, small molecules that inhibit antigen presentation, and even mRNA vaccines [62]. Despite these efforts, the search for a durable and effective T1D cure remains ongoing. To a certain extent, the control of AIDs rely on the ability of T_regs_ to dampen unnecessary immune activation. In recent years, Treg adoptive cell therapy has been shown to be safe in phase 1/2 clinical trials for individuals with T1D. One study conducted in children with T1D demonstrated that C-peptide levels were maintained for more than 2 years [63, 64]. These initiatives hold tremendous potential for advancing T1D therapy, bringing us closer to a future in which insulin may no longer be necessary.

We propose an autologous immunotherapy utilizing immune cells to clear antigen-specific T cells involved in type 1 diabetes pathogenesis. The approach exploits ADCC or CDC mediated by the constant Fc region of antibodies to specifically destroy insulin-reactive CD4^+^ T cells. A key CD4^+^ T cell epitope identified in type 1 diabetes is insulin B chain amino acids 9–23 (InsB9–23). We have engineered a novel reagent, designated Insulin-MHC-Fc, by combining the InsB9-23 peptide, MHC, and the Fc region of human IgG1 antibodies. This unique tripartite complex comprises the InsB9-23 peptide bound to MHC for engagement of the T cell receptor, and the Fc domain to recruit cytotoxic immune cells via ADCC or activate complement via CDC. Standard molecular cloning techniques and recombinant protein expression systems with stringent purification schemes can be utilized to generate the Insulin-MHC-Fc complex with high specificity and affinity for the target T cells. Administration of Insulin-MHC-Fc to type 1 diabetes patients is expected to coat insulin-reactive CD4^+^ T cells via T cell receptor-MHC interaction. The Fc region would then trigger ADCC or CDC mechanisms, leading to depletion of pathogenic insulin-specific T cells. In summary, the engineered Insulin-MHC-Fc complex represents a tailored immunotherapeutic agent capable of utilizing patients’ endogenous immune cells to achieve antigen-specific clearance of autoreactive T cells in type 1 diabetes.

## Perspective

The tremendous advancements in our understanding of the intricate pathophysiology, antigenic specificities and cellular heterogeneity underlying autoimmune disorders in recent years have enabled and motivated the development of novel strategies to selectively remove antigen-specific autoreactive T and B cells. By specifically targeting the pathogenic lymphocytes, these emerging techniques can potentially avoid the side effects of broad immunosuppression caused by current medications. Moreover, eliminating the autoreactive clones may facilitate restoration of immunological homeostasis and stability in a disease-specific manner - a desirable therapeutic outcome that non-antigen-specific approaches struggle to achieve. We believe that in the not-too-distant future, these antigen-targeted therapies will provide patients with improved clinical benefits via tailored immunosuppression focused on the tissue and disease specificity of the pathogenic lymphocytes. The current knowledge of autoimmune pathogenesis sets the stage for translating antigen-specific therapies from conceptual promise to clinical reality. With continued research elucidating the antigenic specificities and immunological nuances in various autoimmune conditions, innovative antigen-directed strategies can be refined to selectively modulate the underlying autoimmunity while minimizing systemic immunosuppression. In summary, recent scientific advances have created substantial optimism that antigen-specific techniques will soon transition from theoretical potential to practical application as breakthrough treatments for autoimmune diseases.
